# Adsorption of Levofloxacin onto Graphene Oxide/Chitosan Composite Aerogel Microspheres

**DOI:** 10.3390/gels10010081

**Published:** 2024-01-21

**Authors:** Pengpai Miao, Jie Gao, Xiaobing Han, Yuan Zhao, Tao Chen

**Affiliations:** 1 School of Pharmacy, Hubei University of Science and Technology, Xianning 437100, China; zzu666666@126.com; 2 School of Nuclear Technology and Chemistry & Biology, Hubei Key Laboratory of Radiation Chemistry and Functional Materials, Hubei University of Science and Technology, Xianning 437100, China; hanxiaobing@hbust.edu.cn (X.H.); zhyf308@hbust.edu.cn (Y.Z.)

**Keywords:** adsorption, levofloxacin, graphene oxide, chitosan, aerogel microspheres

## Abstract

The removal of pharmaceutical residues from water resources using bio-based materials is very important for human safety and health. Bio-based graphene oxide/chitosan (GO/CS) aerogel microspheres were fabricated with emulsification and cross-linking, followed by freeze drying, and were used for the adsorption of levofloxacin (LOF). The obtained GO/CS aerogel microspheres were characterized with scanning electron microscopy (SEM), Fourier-transform infrared (FTIR), and thermogravimetry (TG). The effects of GO content, pH value, and temperature on their adsorption capacity were investigated. With the incorporation of 40 wt% GO, the adsorption capacity increased from 9.9 to 45.6 mg/g, and the highest adsorption capacity, 51.5 mg/g, was obtained at pH = 8 and T = 25 °C. In addition, to obtain deeper insight into the adsorption process, the thermodynamics and kinetics of the process were also investigated with four different models of LOF adsorption. The thermodynamic modeling results revealed that LOF adsorption is exothermic, and the kinetic investigation demonstrated that LOF adsorption is generally consistent with a pseudo-first-order rate law.

## 1. Introduction

With the development of modern industry, many emerging contaminants have been found in water resources. After traditional heavy-metal ions and various types of dyes, pharmaceuticals are the main emerging contaminants [1,2,3]. Among these pharmaceuticals, levofloxacin (LOF) has caused widespread concern. As LOF can be used to treat many bacterial infections, it has been widely used in humans and animals. In addition, LOF is hard to metabolize and exhibits resistance to removal by conventional treatments. Thus, the removal of LOF from water resources is very important for human safety and health [4,5].

To solve the problem of LOF residues in water resources, many materials have been developed for the removal of LOF. For example, metformin-grafted chitosan/poly(vinyl alcohol), a material with superior adsorption properties, was used for the adsorption of LOF [6]; in this system, the abundant hydrogen and chemical bonds increased the surface area of the obtained porous materials to 83.8 m^2^/g. The abundant amino groups originating from metformin improve the adsorption properties dramatically, conferring to the materials an adsorption capacity as high as 2872.927 mg/g. Based on the strong interaction between the metal-organic framework and LOF, ZIF-8 was used for the adsorption of LOF [7], and a high adsorption capacity of 194.1 mg/g) was observed for this material. Mahmoud et al. synthesized nanotitanium oxide/chitosan/nano-bentonite composites and used them for the removal of LOF [8]. These authors obtained a high removal efficiency (92.4%) and an adsorption capacity of 1.54 mg/g. They next prepared vanadium pentoxide@chitosan@MOFs nanobiosorbent [9] and obtained a removal percentage of LOF from wastewater as high as 97.2%, with an adsorption capacity of 1.94 mg/g. Graphene oxide-embedded cellulose nanocrystal composites have also been fabricated and used for the adsorption and removal of LOF [10]. The obtained three-dimensional materials exhibited an adsorption capacity of 8.01 mg/g.

Similarly, owing to their unique structure and properties, graphene oxide (GO) [11,12] and chitosan (CS) [13,14,15] have also been widely used for the removal of contaminants. Due to its aromatic planar structure and its oxygen-containing functional groups, including -OH, -C=O, and -COOH, GO has attracted tremendous scientific interest for use in environmental applications, especially for the adsorption of organic and inorganic pollutants. GO and GO-based composites have been investigated as efficient adsorbents for many contaminants, such as cationic/anionic dyes, phenolics, nitroaromatics, and heavy-metal ions [11]. In addition, GO, reduced GO, and modified GO have also been widely used in the removal of pharmaceuticals from aqueous solutions and have been tested on ciprofloxacin, tetracycline, sulfamethoxazole, diclofenac, carbamazepine, etc. [12]. Due to its abundance and large number of functional groups (-OH and -NH_2_), CS has also been widely used in the removal of pollutants from water resources. The high adsorption capacity of CS-based adsorbents for organic pollutants and heavy metals can be attributed to their large number of adsorption sites, high reactivity, excellent chelation behavior, and flexible structure. Pure CS powder was used directly for the removal of acid blue 9 and yellow 3 from aqueous solution. The maximum adsorption capacities were 210 and 295 mg/g, respectively [13]. Pure CS beads with a diameter of about 1 mm were fabricated for the adsorption of Congo red, and an adsorption capacity of 54.8 mg/g was obtained at room temperature. The adsorption capacity was enhanced by the incorporation of lignin [15]. In addition, CS and CS-based composites were also developed for the removal of heavy-metal ions (Cu, Ni, Cr, Cd, Pd, Hg) and precious-metal ions (Ag and Au) [15].

Combining the unique structures and properties of these materials, biocompatible and biodegradable GO/CS composites have also been extensively investigated and used in different areas [16,17,18]. At an early stage, GO/CS composites were fabricated by glutaraldehyde cross-linking and used for the adsorption of Au(III) and Pd(II) [19]. The maximum adsorption capacities were observed for the 5wt%GO/CS composite, at 1076.65 and 216.92 mg/g for Au(III) and Pd(II), respectively. GO/CS composites have been widely used for the adsorption of dyes. CS/GO aerogel microspheres were fabricated by supercritical drying with CO_2_. A high adsorption capacity (178.25 mg/g) was obtained for bilirubin within 2 h [20]. CS/GO composite hydrogels have also been applied in water purification as broad-spectrum adsorbents that can effectively remove both cationic and anionic dyes [21]. The highest capacities for alkaline methylene blue (MB) and acidic eosin Y were both greater than 300 mg/g. Beyond the removal of dyes [22,23], a CS/GO composite has also been used for the adsorption of protein. A magnetic CS/GO composite was fabricated and used for the adsorption of cytochrome c. The highest adsorption capacity in this system was 13.3 mg/g [24]. However, to date, there are no reports regarding the removal of LOF with a CS/GO composite.

**Figure 1:**
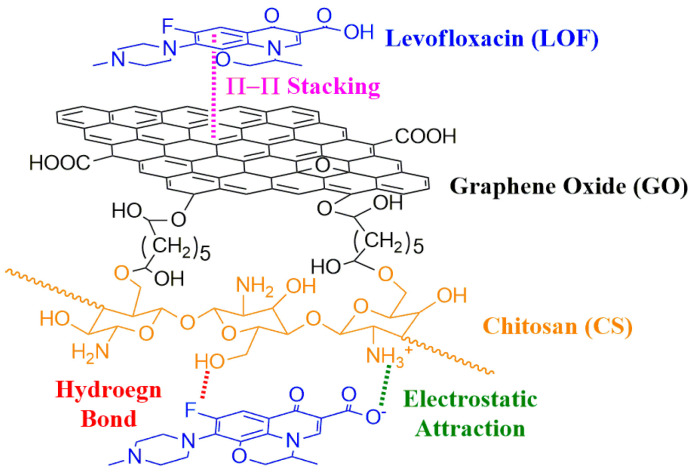
GO/CS aerogel microspheres for the removal of LOF by adsorption.

As mentioned above, based on the various possible intermolecular interactions (hydrogen bonding, π-π stacking interactions, electrostatic attraction), biocompatible and biodegradable GO/CS aerogel microspheres were fabricated and used for the adsorption of LOF (Figure 1). The GO/CS microspheres were fabricated by chemical cross-linking and freeze-drying, and the adsorption properties and kinetics of LOF adsorption were also investigated.

## 2. Results and Discussion

### 2.1. Characterization of GO/CS Aerogel Microspheres

#### 2.1.1. SEM Analysis

**Figure 2:**
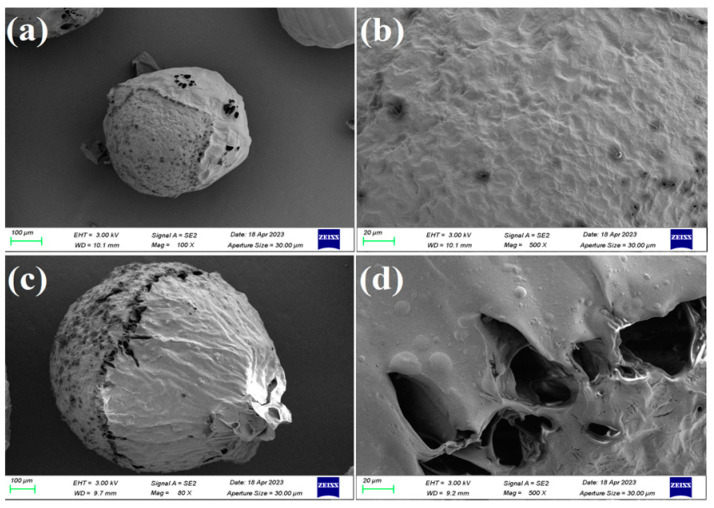
SEM images of CS microspheres (**a**,**b**) and 40%GO/CS microspheres (**c**,**d**).

The morphology of the CS aerogel microspheres and 40%GO/CS aerogel microspheres was characterized with SEM. The results are shown in Figure 2. The aerogel microspheres fabricated by freeze-drying exhibit a porous structure, which will facilitate the adsorption of LOF. This structure is quite different from that of the microspheres obtained via traditional hot-drying, which have a dense structure [25,26,27]. This difference demonstrates that freeze-drying is an useful technique for the preparation of porous materials. In addition, the GO/CS microspheres are larger in size and have more holes than the CS microspheres, showing that the introduction of GO dramatically changes the structure of the obtained microspheres.

#### 2.1.2. FTIR Analysis

**Figure 3:**
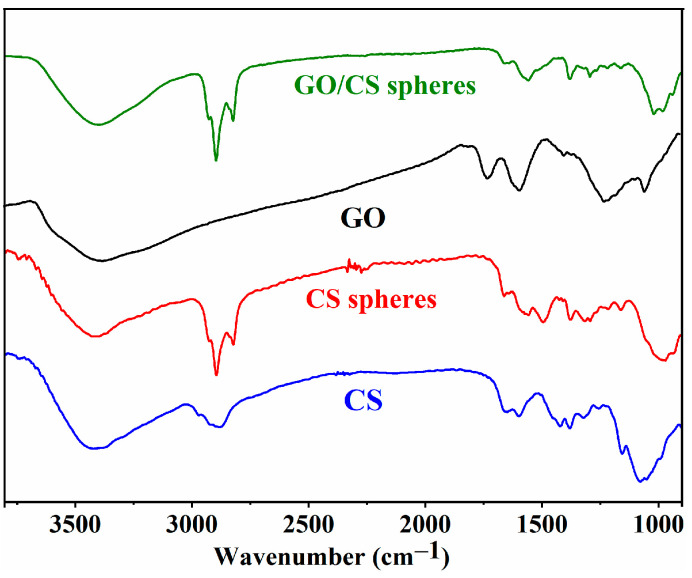
FTIR spectra of CS, CS microspheres, GO, and 40%GO/CS microspheres.

The composition of raw CS, CS aerogel microspheres, GO, and 40%GO/CS aerogel microspheres was analyzed with FTIR spectroscopy. The results are shown in Figure 3. The FTIR spectra of raw CS show obvious peaks at 3200–3600 cm^−1^ (-OH, -NH_2_, and intermolecular hydrogen bonds), 2930 and 1375 cm^−1^ (C-H), 1650 cm^−1^ (C-N), 1160 cm^−1^ (C-OH), 1090 cm^−1^ (C-O-C) [28,29]. In the spectra generated for the CS aerogel microspheres, two new peaks at 2861 cm^−1^ (methylene), and 1690 cm^−1^ (C=N) were observed, indicating chemical cross-linking with glutaraldehyde [29,30]. The FTIR spectra of GO show peaks at 3400 cm^−1^ (-OH), 1723 cm^−1^ (C=O), 1600 cm^−1^ (C=C), and 1050 cm^−1^ (C-O) [5,22]. The spectra of the GO/CS aerogel microspheres confirmed the successful fabrication of GO/CS aerogel microspheres, as all of the characteristic peaks of CS, GO, and glutaraldehyde appeared [15,30]. The abundant -OH and -NH_2_ groups of GO/CS will easily form hydrogen bonds with nitrogen, oxygen, and fluorine atoms, all of which are present in the structure of LOF. In addition, it can be inferred that -NH_2_ originating from CS will be protonated to -NH_3_^+^ by -COOH originating from LOF and that there is an electrostatic interaction between CS and LOF [15,28]. Furthermore, a π-π stacking interaction can also form between the aromatic structure of GO and LOF [25,26]. All of these intermolecular interactions will improve the adsorption performance of the obtained GO/CS aerogel microspheres.

#### 2.1.3. TG Analysis

**Figure 4:**
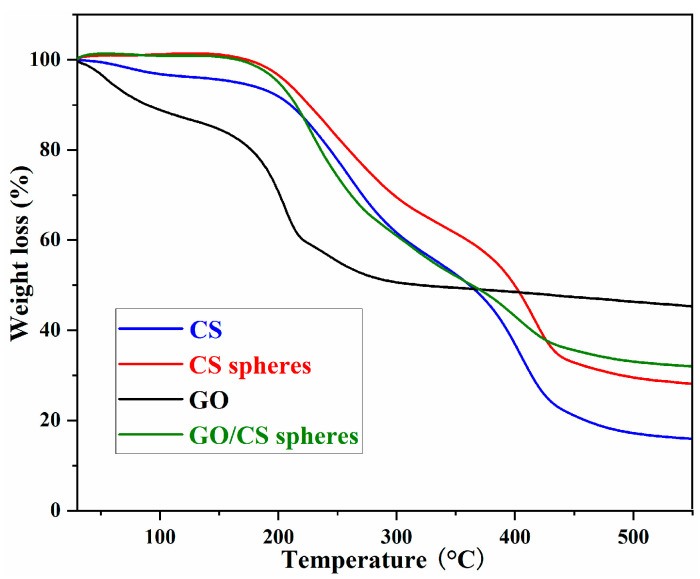
TG curves of CS, CS microspheres, GO, and 40%GO/CS microspheres.

The thermal stability of raw CS, CS aerogel microspheres, GO, and 40%GO/CS aerogel microspheres was investigated by thermogravimetric analysis. The results are shown in Figure 4. The thermal degradation of raw CS proceeded in three stages: the 4.2wt% weight loss before 150 °C can be ascribed to the absorbed water; the 36.6wt% weight loss between 180 to 320 °C is due to the loss of side groups; and the weight loss after 320 °C is ascribed to the degradation of main chain [23,28]. Compared with raw CS, the thermal stability of CS aerogel microspheres was significantly better, which can be ascribed to the chemical cross-linking with glutaraldehyde [15,23]. The thermal degradation of GO proceeded in two stages: the 8.6wt% weight loss before 150 °C can also be ascribed to the absorbed water, and the weight loss after 200 °C is due to the degradation of oxygen-containing groups [5,31]. With the incorporation of GO, the GO/CS microspheres exhibit improved thermal stability, but their thermal stability is still less than that of CS microspheres, a difference that can be attributed to the bad thermal stability of GO [15,23].

### 2.2. Adsorption Performance of GO/CS Aerogel Microspheres

Levofloxacin (LOF) is a broad-spectrum antibiotic whose structure contains nitrogen, oxygen, and fluorine atoms, negatively charged groups (-COOH), and aromatic rings (quinolones) [32]. As mentioned in the description of the FTIR analysis, the obtained GO/CS composites can easily form hydrogen bonds, electrostatic attraction, and π-π stacking interactions with LOF [15,33,34]. The fabricated GO/CS aerogel microspheres were used for the removal of LOF with variations in experimental parameters, such as GO content, solution pH value, temperature, initial concentration, and contact time.

#### 2.2.1. Effect of GO Content and pH Value

**Figure 5:**
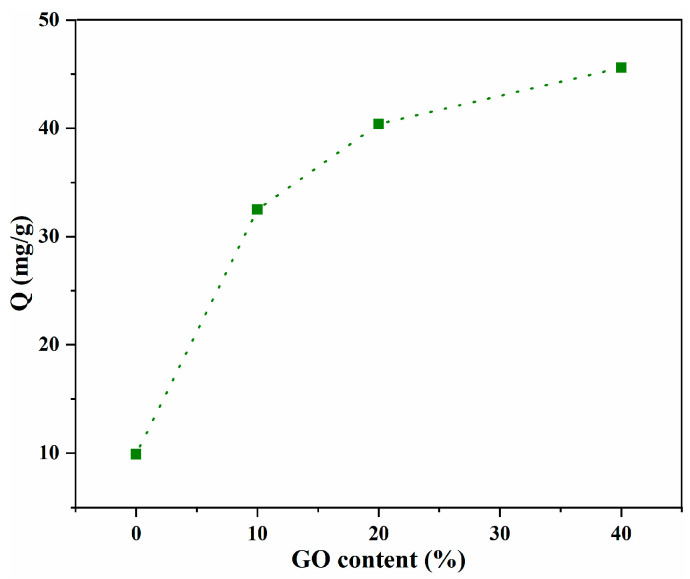
Influence of GO content on the adsorption capacity.

The influence of GO content on the capacity of the material to adsorb LOF was investigated first, and the results are presented in Figure 5. Compared to the adsorption capacity of pure CS microspheres (9.9 mg/g), the incorporation of GO clearly improves the adsorption performance of GO/CS, and the adsorption capacity is highly dependent on the content of GO [23]. In addition, the maximum adsorption capacity, 45.6 mg/g, was obtained with the aerogel microspheres containing 40%GO. As the GO content increased, the rate of increase in LOF adsorption capacity slowed, an effect of LOF diffusion being hindered by exceedingly rigid GO and cross-linking [23,26]. Taking the cost of GO and enhanced efficiency into account, GO/CS composites with higher GO contents were not investigated, and 40%GO/CS aerogel microspheres were chosen for further study.

**Figure 6:**
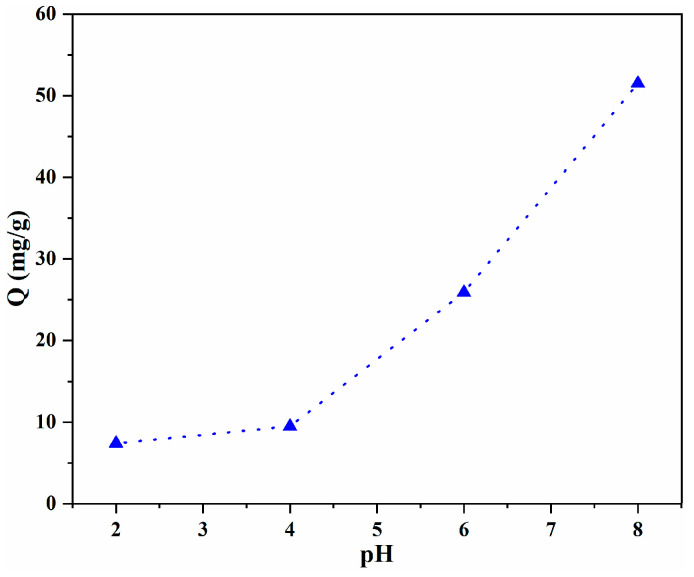
Influence of pH value on the adsorption capacity.

The pH value of the solution has a significant effect on the adsorption of LOF. Thus, the effect of pH was investigated, and the results are shown in Figure 6. The adsorption of LOF by 40%GO/CS shows high dependence on the pH value, revealing that electrostatic attraction is an important factor in the adsorption process. As the pH value increased, the adsorption capacity increased significantly, and the greatest adsorption capacity, 51.5 mg/g, was observed at pH = 8. This relationship can be ascribed to the different charges of LOF and GO/CS microspheres at different pH values [15,25]. LOF presents as a zwitterionic molecule at pH values between 6 and 8, possesses a positive charge at pH ˂ 6, and is negatively charged at pH > 8 [35,36]. Thus, extensive repulsion exists between positive LOF and -NH_3_^+^ (CS moiety) when the pH ˂ 6, reducing the adsorption of LOF at low pH values. As pH value increases, the repulsion is reduced and the adsorption capacity is correspondingly enhanced, with the greatest adsorption obtained at pH = 8. As reported in the literature [35,36], when the pH value is increased further, the negatively charged LOF has a weak electrostatic attraction with neutral -NH_2_ (CS moiety), but has a repulsive interaction with negatively charged -COO^-^ (GO moiety); thus, the adsorption of LOF will decrease at pH > 8. For this reason, higher pH values were not investigated and all further investigations were conducted at a pH of 8.

#### 2.2.2. Effect of Temperature and Thermodynamics

**Figure 7:**
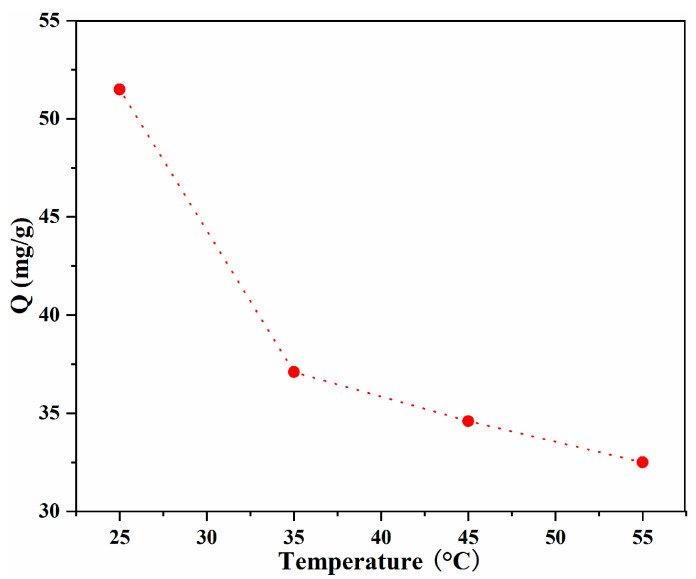
Influence of temperature on the adsorption capacity.

In addition to the pH value, temperature is another key parameter that has a significant influence on adsorption. The influence of temperature on the adsorption of LOF by 40%GO/CS was studied, and the results are shown in Figure 7. As can be clearly seen from the figure, the adsorption capacity decreased as the temperature increased, and the maximum capacity was observed at 25 °C. This result demonstrates that the adsorption process is exothermic [37,38]. Although low temperatures can increase adsorption capacity, upon considering the energy consumption needed for lower-temperature investigations and the accessibility of equipment, further investigations were conducted at 25 °C.

Thermodynamic investigations are very important in elucidating LOF adsorption, as the data produced can reveal whether the adsorption process is endothermic or exothermic and whether it is spontaneous. The thermodynamic parameters Gibbs free energy change (Δ*G*^0^), enthalpy change (Δ*H*^0^), and entropy change (Δ*S*^0^) were calculated using Equations (1)–(3) [26,37]:

*lnK*^0^ = *ln*(*Q_e_*/*C_e_*) (1)

Δ*G*^0^ = −*RT ln K*^0^ (2)

*Rln K*^0^ = −Δ*H*^0^/*T* + Δ*S*^0^ (3)

where *Q_e_* is the adsorption capacity at equilibrium and *C_e_* is the equilibrium concentration, *R* is 8.314 J/mol·K. The enthalpy change (Δ*H*^0^) and entropy change (Δ*S*^0^) were calculated from the plot of *lnK*^0^ versus 1/*T*.

Currently, there are two approaches used to calculate the equilibrium constant *K*^0^ in thermodynamic evaluations. In the first approach, the equilibrium constant *K*^0^ is calculated from *Q_e_* and *C_e_*, which are obtained from the investigation of effect of temperature on the adsorption capacity (Equation (4)) [7,8,9,19,39,40]. As illustrated in the cited works, the influence of temperature on the thermodynamic parameters is related to the adsorption process. The equilibrium constant *K*^0^ can be calculated as *Q_e_*/*C_e_*, and the values of *Q_e_* and *C_e_* are obtained at different temperatures and with a specific initial concentration.

*K*^0^ = *Q_e_*/*C_e_* (4)

In the other approach, the equilibrium constant *K*^0^ is derived from the best-fitted isotherm adsorption models (Equation (5)) [41,42,43,44,45], such as the Langmuir model (which assumes a monolayer and homogeneous adsorption onto a surface with no interaction between the adsorbate molecules) and the Freundlich model (which assumes multilayer adsorption onto heterogeneous surfaces with interaction between the adsorbate molecules) [14,23]. Adsorption isotherms can elucidate the interaction between the absorbent and the absorbate at equilibrium. These models are always created at single specified temperatures with varied initial concentrations. Next, different adsorption isotherm models were tested against real adsorption data, and *K*^0^ of the best-fitted model was used to calculate thermodynamic parameters.

*K*^0^ = *K_L_*_/*F*_ × 1000 × molecular weight of adsorbate (5)

Based on the results regarding the effect of temperature, the thermodynamics of LOF adsorption onto GO/CS aerogel microspheres were investigated through the first approach, and the obtained results are listed in Table 1. As shown in Table 1, with the increase in temperature, the Δ*G*^0^ increased from −0.51 to −0.33 kJ/mol, revealing that the adsorption process is spontaneous. This result also indicated that low temperatures are beneficial for the adsorption of LOF, a finding that is consistent with the results for the effect of temperature on adsorption [37,38]. In addition, the Δ*H*^0^ and Δ*S*^0^ were −2.21 kJ/mol and −5.8 J/mol·K, respectively. The negative value of Δ*H*^0^ shows that LOF adsorption is exothermic, in agreement with other data, and the positive value of Δ*S*^0^ shows that GO/CS aerogel microspheres have good affinity for LOF [26,37].

#### 2.2.3. Effect of Concentration, Time and Kinetics

**Figure 8:**
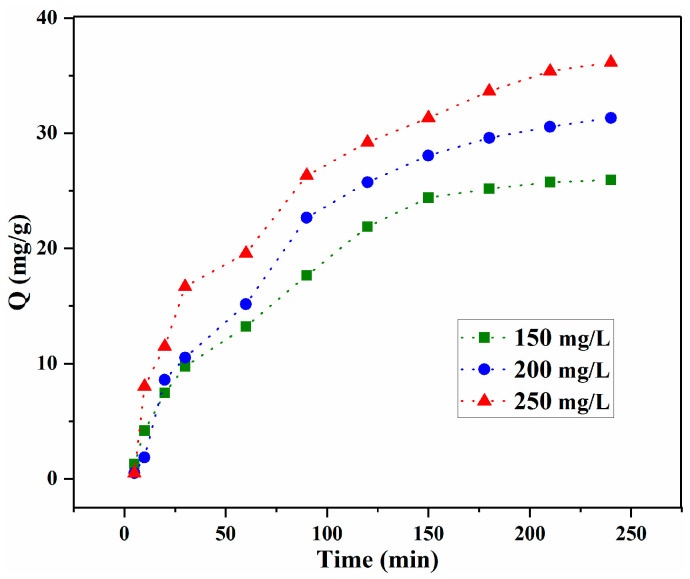
Influence of concentration and time on adsorption capacity.

The influence of contact time on LOF adsorption onto GO/CS aerogel microspheres was assessed at different concentrations (150, 200, 250 mg/g), and the results are shown in Figure 8. The results indicate higher adsorption capacity and a longer time to equilibrium at high concentrations, demonstrating that adsorption is highly dependent on concentration [37,46,47].

To obtain deep insight into the adsorption of LOF onto GO/CS microspheres, based on the results regarding the effects of concentration and contact time, the adsorption kinetics were investigated. The most-used kinetic models are pseudo-first-order (PFO) and pseudo-second-order (PSO) models. In the PFO model, the rate-limiting step is diffusion of adsorbate molecules, while the PSO model assumes that the process is controlled by the adsorption reaction at the interface of adsorbents [48]. Beyond the PFO and PSO models, Elovich and intraparticle diffusion (IPD) have also been widely used in the kinetic investigation of adsorption [26,37]. The Elovich model is also suitable for chemical adsorption with a heterogeneous absorbing surface, especially for chemical adsorption on a solid surface without desorption and an adsorption rate that decreases with increased surface coverage [49]. In the IPD model, the adsorption on the external surface is the first step; the second step is intraparticle-diffusion-controlled, gradual adsorption; the final step is the slow movement of the adsorbate from larger pores to micropores [50].

Based on the findings with regard to the effects of concentration and contact time, the PFO, PSO, Elovich, and IPD kinetic models were used for the evaluation of LOF adsorption (Equations (6)–(9)) [15,26,37].

PFO model:

(6) $$ln(Q_{e}-Q_{t})=lnQ_{e}-k_{1}t$$

PSO model:

(7) $$\frac{t}{Q_{t}}=\frac{1}{k_{2}Q_{e}^{2}}+\frac{t}{Q_{e}}$$

Elovich model:

(8) $$Q_{t}=\frac{ln\alpha \beta }{\beta }+\frac{1}{\beta }lnt$$

IPD model:

(9) $$Q_{t}=k_{p}t^{1/2}+C$$

where *Q_e_* is the adsorption capacity at equilibrium; *Q_t_* is the adsorption capacity at *t*; *k*_1_, *k*_2_, *k_p_* is the rate constants for PFO, PSO, and IPD models; *α* is the initial rate of adsorption; *β* is the constant of desorption; and *C* is a constant.

**Figure 9:**
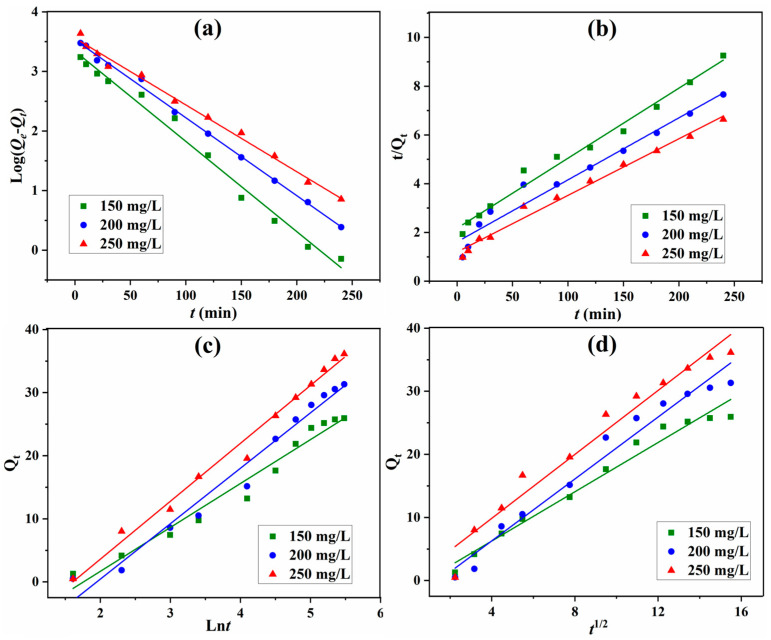
Fitting of PFO (**a**), PSO (**b**), Elovich (**c**), and IPD (**d**) kinetic models for the adsorption of LOF onto 40%GO/CS.

The fitting curves of adsorption by GO/CS microspheres with the PFO, PSO, Elovich, and IPD models are presented in Figure 9. The corresponding kinetic parameters were calculated and are listed in Table 2. For the adsorption capacity at equilibrium with different concentrations, the calculated value is quite close to the experimental one for the PFO kinetic model. All of the kinetic models have a *R*^2^ coefficients greater than 0.96, indicating that different modes of LOF adsorption occur concurrently. The highest coefficient values (0.9885, 0.9978, 0.9933) were obtained at different concentrations with the PFO model, indicating that this model fits the adsorption process better than others. Thus, PFO is the predominant mode of LOF adsorption onto GO/CS aerogel microspheres and diffusion is the rate-limiting step in LOF adsorption [15,26,37].

### 2.3. Comparative Investigation of LOF Adsorption

The performance of the obtained GO/CS aerogel microspheres in the adsorption of LOF was compared with those of similar absorbents. As shown in Table 3, CS/PVA-Pr-Met [6] and ZIF-8 [7] show much higher capacities to adsorb LOF, a difference that can be ascribed to complex chemical modifications or the characteristics of the expensive raw materials, which make these adsorbents unsuitable for use at large scales. Although Nbent-NtiO_2_-Chit [8] and V_2_O_5_@Ch/Cu-TMA [9] composites have very high removal efficiency (92.4% and 97.2%), the amount of absorbent needed is very high, so the true adsorption capacity is low. A similar bio-based adsorbent, CNCs-GO [10], has a much lower adsorption capacity than GO/CS aerogel microspheres, a difference that can be attributed to the weak electrostatic attraction between CNCs and LOF. It can be seen that the adsorption capacity of GO/CS is better than those of most other absorbents. It is a low-cost, biocompatible and biodegradable material with no metal component that shows promise for application in the removal of pharmaceuticals from water resources.

## 3. Conclusions

In summary, bio-based GO/CS aerogel microspheres were successfully fabricated via emulsification, cross-linking, and freeze-drying, and the obtained porous microspheres were used for the efficient removal of LOF. The SEM images showed that GO/CS microspheres are larger and have more holes than CS microspheres, and the TG analysis indicated that cross-linking can enhance the thermal stability of the obtained composite microspheres. The adsorption experiments revealed that the optimal conditions for adsorption are pH = 8 and T = 25 °C, and the highest adsorption capacity, 51.5 mg/g, was obtained with 40%GO/CS aerogel microspheres. According to the FTIR analysis, there are three types of interactions between GO/CS microspheres and LOF: hydrogen bonds, electrostatic interactions, and π-π stacking interactions. The effect of pH on the adsorption capacity confirms that the electrostatic attraction between GO/CS microspheres and LOF has a significant effect on adsorption. In addition, the thermodynamic data indicate that the adsorption process is exothermic and spontaneous, and the kinetic data reveal that diffusion is the rate-limiting step. To further improve this investigation, future work will focus on scaled-up production, dynamic column adsorption, and selectivity enhancement.

## 4. Materials and Methods

### 4.1. Materials

Graphene oxide (GO, 98%) was supplied by Suzhou TanFeng Co., Ltd. (Suzhou, China). Acetic acid (99.5%), chitosan (CS, deacetylation degree > 95%), glutaraldehyde (50%), and levofloxacin (LOF, ≥98%) were purchased from HWRK Chemical Co., Ltd. (Beijing, China). Liquid paraffin, petroleum ether, and Tween-80 were provided by Sinopharm Chemical Reagent Co., Ltd. (Shanghai, China). Distilled water was used throughout the experiments for sample and solution preparation.

### 4.2. Fabrication of GO/CS Aerogel Microspheres

The GO/CS aerogel microspheres were fabricated as follows [15]: GO powder (0, 10, 20, 40wt%) was dispersed into water with a bath sonicator for half an hour. Acetic acid and the corresponding weight of CS were added to the GO dispersion under vigorous stirring at 50 °C. Then, the mixture was poured into the emulsifier solution, which consisted of liquid paraffin, petroleum ether, and Tween-80. Half an hour later, glutaraldehyde was added dropwise for cross-linking. One hour later, GO/CS hydrogel microspheres were obtained by filtration and washing. The GO/CS aerogel microspheres were then fabricated by freeze-drying.

### 4.3. Characterization of GO/CS Aerogel Microspheres

Fourier-transform infrared spectra were obtained on an Avatar 360 Nicolet instrument (Thermo Fisher Scientific, Shanghai, China) using KBr pellets with wave numbers from 4000 to 40 cm^−1^ and a resolution of 4 cm^−1^. UV-Vis absorption spectra were obtained with a S 3100 spectrophotometer (Mapada Instruments Co., Ltd., Shanghai, China). Thermogravimetry analysis was conducted using a NETZSCH TG 209F3 instrument (NETZSCH Scientific Instruments Trading, Ltd., Shanghai, China) under an N_2_ atmosphere, with a heating rate of 10 °C/min from ambient temperature to 700 °C. The morphologies were analyzed by scanning electron microscopy (SEM, VEGA-3, Tescan, Czech Republic).

### 4.4. Adsorption of GO/CS Aerogel Microspheres

#### 4.4.1. Calibration Plot of LOF

The absorbance of LOF at different concentrations was measured using UV-Vis absorption spectroscopy at 291 nm [6,8]. The correlation of absorbance and concentration is *A* = 0.0692 *C* + 0.0126, with a coefficient of 0.9981.

#### 4.4.2. Effect of GO Content, pH Value, and Temperature

Adsorption experiments were conducted in a 100 mL flask, with 30 mg GO/CS aerogel microspheres added into 30 mL LOF solution at a concentration of 300 mg/L. Then, the mixture was placed in a shaking incubator for 24 h with a speed of 150 rpm. The adsorption capacity *Q* (mg/g) was calculated using Equation (8).

*Q* = (*C*_0_ − *C_t_*)V/m (10)

where *C*_0_ is the initial concentration; *C_t_* is the final concentration; m is the weight of GO/CS aerogel microspheres; and V is the volume of the solution.

#### 4.4.3. Effect of Initial Concentration and Contact Time

To reveal the kinetics of LOF adsorption onto GO/CS aerogel microspheres, the influence of initial concentration and contact time were investigated. Following a standard procedure, the adsorption of LOF with different contact times was measured at different LOF concentrations (150, 200, and 250 mg/L), and the adsorption capacity was determined and calculated at predetermined intervals within 4 h.

## Figures and Tables

**Table 1:** Thermodynamic parameters for the adsorption of LOF.

Temperature (K)	*K* ^0^	Δ*G*^0^ (kJ/mol)	Δ*H*^0^ (kJ/mol)	Δ*S*^0^ (J/mol·k)
298	0.21	−0.51	−2.21	5.8
308	0.14	−0.36	-	-
318	0.13	−0.34	-	-
328	0.12	−0.33	-	-

**Table 2:** Kinetic results of different models of LOF adsorption.

Kinetic Models	Coefficients	150 mg/L	200 mg/L	250 mg/L
PFO	*q*_e,cal_ (mg/g)	28.22	34.12	35.16
	*k*_1_ (min^−1^)	1.51 × 10^−2^	1.31 × 10^−2^	1.12 × 10^−2^
	*R* ^2^	0.9885	0.9978	0.9933
PSO	*q*_e,cal_ (mg/g)	34.84	39.31	43.05
	*k*_2_ (g/mg min)	3.79 × 10^−4^	4.00 × 10^−4^	4.50 × 10^−4^
	*R* ^2^	0.9851	0.9630	0.9880
Elovich	*A* (mg/g min)	1.1959	1.2502	1.8356
	*Β* (g/min)	0.1437	0.1138	0.1089
	*R* ^2^	0.9688	0.9732	0.9883
IPD	*K_p_* (g/kg min^1/2^)	1.9528	2.4548	0.5350
	*C* (g/kg)	−1.5524	−3.5384	−0.2857
	*R* ^2^	0.9758	0.9720	0.9642

**Table 3:** Comparison of LOF adsorption by different adsorbents.

Absorbents	Qmax (mg/g)	Reference
GO/CS	51.5	This work
CS/PVA-Pr-Met	2872.9	[6]
ZIF-8	194.1	[7]
Nbent-NtiO_2_-Chit	1.54	[8]
V_2_O_5_@Ch/Cu-TMA	1.94	[9]
CNCs-GO	8.01	[10]

## Data Availability

All data and materials are available on request from the corresponding author. The data are not publicly available due to ongoing research using a part of the data.
